# Predicting severe outcomes using national early warning score (NEWS) in patients identified by a rapid response system: a retrospective cohort study

**DOI:** 10.1038/s41598-021-97121-w

**Published:** 2021-09-09

**Authors:** Sang Hyuk Kim, Hye Suk Choi, Eun Suk Jin, Hayoung Choi, Hyun Lee, Sang-Hwa Lee, Chang Youl Lee, Myung Goo Lee, Youlim Kim

**Affiliations:** 1 Division of Pulmonology and Critical Care Medicine, Department of Medicine, Samsung Medical Center, Sungkyunkwan University School of Medicine, Seoul, Korea; 2 Department of Rapid Response Team, Advanced Practice Nurse, Hallym University Chuncheon Sacred Heart Hospital, Chuncheon, Korea; 3 Division of Pulmonary, Allergy, and Critical Care Medicine, Department of Internal Medicine, Hallym University Kangnam Sacred Heart Hospital, Hallym University College of Medicine, Seoul, Korea; 4 Division of Pulmonary Medicine and Allergy, Department of Internal Medicine, Hanyang University College of Medicine, Seoul, Korea; 5 Department of Neurology, Hallym University Chuncheon Sacred Heart Hospital, Hallym University College of Medicine, Chuncheon, Korea; 6 Division of Pulmonary, Allergy and Critical Care Medicine, Department of Internal Medicine, Hallym University Chuncheon Sacred Heart Hospital, Hallym University College of Medicine, 77, Sakju-ro, Chuncheon, Gangwon-do 24253 Korea

**Keywords:** Outcomes research, Risk factors

## Abstract

There are insufficient data in managing patients at high risk of deterioration. We aimed to investigate that national early warning score (NEWS) could predict severe outcomes in patients identified by a rapid response system (RRS), focusing on the patient’s age. We conducted a retrospective cohort study from June 2019 to December 2020. Outcomes were unplanned intensive care unit (ICU) admission, ICU mortality, and in-hospital mortality. We analyzed the predictive ability of NEWS using receiver operating characteristics (ROC) curve and the effect of NEWS parameters using multivariable logistic regression. A total of 2,814 RRS activations were obtained. The predictive ability of NEWS for unplanned ICU admission and in-hospital mortality was fair but was poor for ICU mortality. The predictive ability of NEWS showed no differences between patients aged 80 years or older and under 80 years. However, body temperature affected in-hospital mortality for patients aged 80 years or older, and the inverse effect on unplanned ICU admission was observed. The NEWS showed fair predictive ability for unplanned ICU admission and in-hospital mortality among patients identified by the RRS. The different presentations of patients 80 years or older should be considered in implementing the RRS.

## Introduction

The United Nations defines a super-aged society as a society where 20% of the total population is 65 years or older^1^. Population worldwide is rapidly aging and, by 2050, more than 20% of the world’s population is expected to be 65 years or older^2,3^. Including South Korea, one of the world’s fastest aging societies, many countries transformed into a super-aged society^4,5^. Following the current trends, the management of the elderly population is emerging as an important social issue^6^. Elderly patients have various comorbidities, and often cognitive functions become impaired^7,8^. Also, managing elderly patients who require hospitalization is challenging^9–11^. Consequently, early recognition of the patient’s deterioration becomes a crucial part of successful treatment for elderly hospitalized patients^12^.

A rapid response system (RRS) is a preventive system for the early detection of hospitalized patients at high risk of deterioration^13^. Various early warning scoring system was used in the implementation of the RRS^14,15^. The National early warning score (NEWS), created in 2012 by the Royal College of Physicians of London standardizing the assessment of acute illness severity, is one of the early warning scoring systems used to implement RRS^16,17^. Several studies have demonstrated that NEWS effectively detects patients at high risk of deterioration relating to unexpected intensive care unit (ICU) admission and in-hospital mortality^18^. However, in the resource-limited environment such as rural areas of the super-aged society, prioritization of management may be required among patients identified by the RRS^19^. Nonetheless, there was insufficient information that clinicians can use as a reference in their management.

To the best of our knowledge, the usefulness of NEWS was unknown in the management of elderly patients identified by RRS. We hypothesized that NEWS could be used for predicting severe outcomes in managing hospitalized elderly patients at high risk of deterioration. Therefore, the purpose of this study was to investigate the predictive ability of NEWS for severe outcomes in patients identified by the RRS, focusing on the patient’s age.

## Results

### Baseline characteristics of the study population

A total of 2,814 RRS activations from 851 patients were recorded, at an average of 3.3 RRS activations per patient. The mean ± standard deviation (SD) age of all patients was 72.6 ± 14.2, and the proportion of men was 60.2%. The mean ± SD age was 65.1 ± 12.6 for patients 80 years or older and 85.3 ± 4.2 for patients under 80 years, respectively. Baseline characteristics according to age group are depicted in Table 1. In patients under 80 years, the proportions of males, high body mass index (BMI), high systolic blood pressure score, and high heart rate score were higher than patients 80 years or older. For disease classification, differences in diagnosed disease distributions by age group were statistically significant except for gastrointestinal diseases. Pulmonary disease was the most common diagnosis in both groups, while the cardiovascular disease was the least diagnosed in patients under 80 years, and neurological disease was the least diagnosed in patients 80 years or older. Regarding severe outcomes, the proportion of unplanned ICU admissions (10.9% vs. 10.6%, *p* = 0.887) did not differ between the age groups, but proportions of ICU (1.3% vs. 2.9%, *p* = 0.005) and in-hospital mortality (47.5% vs. 40.2%, *p* < 0.001) were higher in patients 80 years or older.

**Table 1:** Baseline characteristics of the study population by age group.

	Age < 80 (n. of RRS activation = 1768, n. of patients = 513)	Age ≥ 80 (n. of RRS activation = 1046, n. of patients = 338)	*p* value
Age	68 (59–75)	84 (82–87)	
**Sex**			
Male	1110 (62.8)	585 (55.9)	0.001
Female	658 (37.2)	461 (44.1)	
**Body mass index (kg/m**^**2**^**)**			
< 21.5	950 (48.9)	511 (51.1)	0.014
≥ 21.5	818 (53.7)	535 (46.3)	
**Respiratory rate (rates/minute)**			
12–20 (0)	458 (25.9)	253 (24.2)	0.396
9–11 (1)	9 (0.5)	3 (0.3)	
21–24 (2)	742 (42.0)	432 (41.3)	
≤ 8 or ≥ 25 (3)	559 (31.6)	358 (34.2)	
**Saturation of percutaneous oxygen (%)**			
≥ 96 (0)	359 (20.3)	197 (18.8)	0.127
94–95 (1)	458 (25.9)	239 (22.8)	
92–93 (2)	349 (19.7)	228 (21.8)	
≤ 91 (3)	602 (34.0)	382 (36.5)	
**Body temperature (°C)**			
36.1–38.0 (0)	1375 (77.8)	834 (79.7)	0.221
35.1–36.0 or 38.1–39.0 (1)	332 (18.8)	182 (17.4)	
≥ 39.1 (2)	47 (2.7)	18 (1.7)	
≤ 35 (3)	14 (0.8)	12 (1.1)	
**Systolic blood pressure (mmHg)**			
111–219 (0)	445 (25.2)	261 (25.0)	0.009
101–110 (1)	305 (17.3)	209 (20.0)	
91–100 (2)	568 (32.1)	364 (34.8)	
≤ 90 or ≥ 220 (3)	450 (25.5)	212 (20.3)	
**Heart rate (beats/minutes)**			
51–90 (0)	299 (16.9)	301 (28.8)	< 0.001
41–50 or 91–110 (1)	680 (38.5)	380 (36.3)	
111–130 (2)	600 (33.9)	279 (26.7)	
≤ 40 or ≥ 131 (3)	189 (10.7)	86 (8.2)	
**Supplemental oxygen**			
Yes	1569 (88.7)	945 (90.3)	0.206
No	199 (11.3)	101 (9.7)	
**Level of consciousness**			
Awake	1 (0.01)	2 (0.02)	0.560
Verbal, pain, or unresponsive	1767 (99.9)	1044 (99.8)	
**Disease classification (reason for hospital admission)**			
Cardiovascular disease	77 (4.4)	97 (9.3)	< 0.001
Pulmonary disease	600 (33.9)	448 (42.8)	< 0.001
Gastrointestinal disease	134 (7.6)	78 (7.5)	0.964
Genitourinary disease	129 (7.3)	111 (10.6)	0.003
Neurological disease	133 (7.5)	40 (3.8)	< 0.001
Cancer	540 (30.5)	126 (12.0)	< 0.001
Other diseases	155 (8.8)	146 (14.0)	< 0.001

Data are expressed as numbers (percentages) except age. Age is expressed as median and interquartile ranges.

### Predicting severe outcomes using NEWS

**Figure 1:**
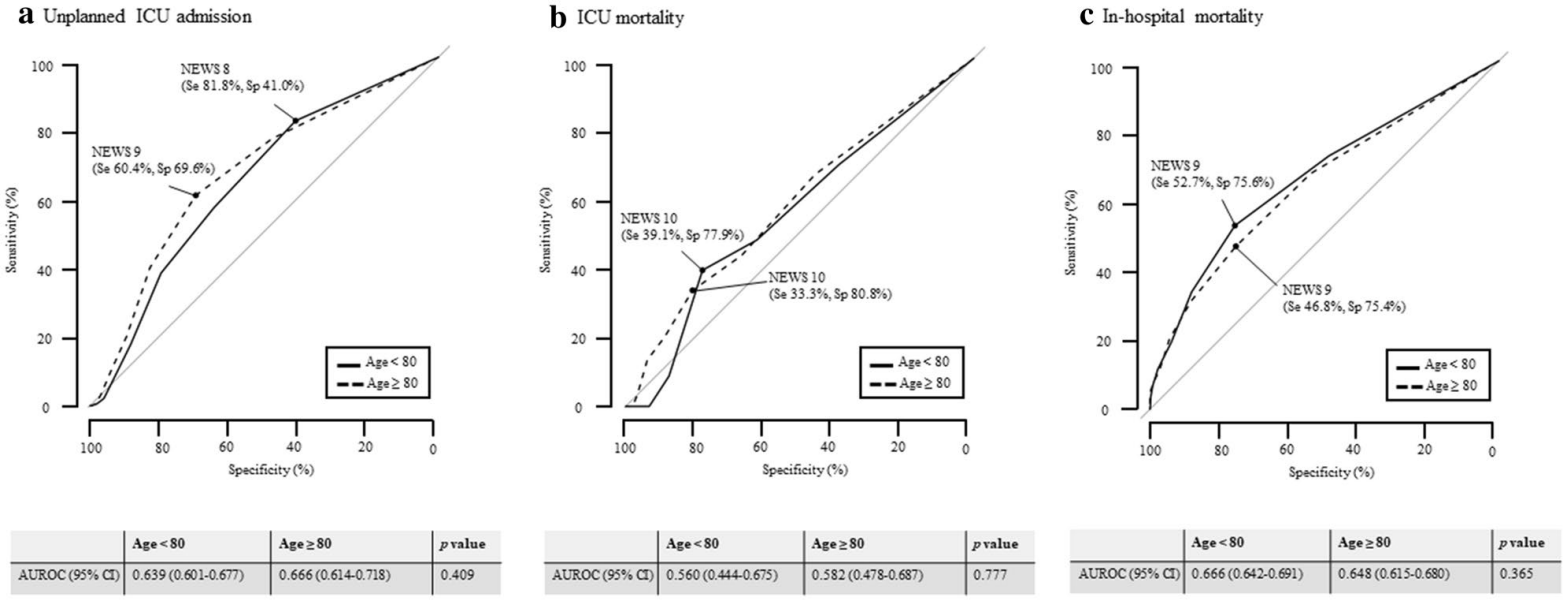
Receiver operating characteristic curves of the National early warning score for severe outcomes by age group. Dots indicate thresholds of National early warning score for the highest accuracy. *NEWS* National early warning score, *ICU* intensive care unit, *AUROC* area under the receiver operating characteristic curve, *Se* sensitivity, *Sp* specificity.

Efficiency NEWS curves for severe outcomes by age group are shown in Supplementary Figure S1. The predictive ability of NEWS for unplanned ICU admission was slightly significantly efficient in patients 80 years or older, but there were no significant differences in the ability to predict ICU and in-hospital mortality. In the ROC curves, the predictive ability of NEWS for unplanned ICU admission (area under the receiver operating characteristic curve [AUROC] 0.650, 95% confidence interval [CI] 0.619–0.680) and in-hospital mortality (AUROC 0.661, 95% CI 0.641–0.680) was fair but was poor for ICU mortality (AUROC 0.566, 95% CI 0.489–0.643). There was no statistically significant difference between patients under 80 years and patients 80 years or older in the comparison of the predictive ability of NEWS for unplanned ICU admission (AUROC 0.639 [95% CI 0.601–0.677] vs. 0.666 [0.614–0.718], *p* = 0.409), for ICU mortality admission (AUROC 0.560 [95% CI 0.444–0.675] vs. 0.582 [0.478–0.687], *p* = 0.777), and for in-hospital mortality admission (AUROC 0.666 [95% CI 0.642–0.691] vs. 0.648 [0.615–0.680], *p* = 0.365) (Fig. 1). In patients 80 years or older, the NEWS cut-off values were 9 for unplanned ICU admission, 10 for ICU mortality, and 9 for in-hospital mortality, respectively. Sensitivity and specificity for severe outcomes according to NEWS are described in Table 2.

**Table 2:** Accuracy of the National early warning score for severe outcomes in high-risk patients by age group.

	NEWS	Unplanned ICU admission		ICU mortality		In-hospital mortality	
		Sensitivity, % (95% CI)	Specificity, % (95% CI)	Sensitivity, % (95% CI)	Specificity, % (95% CI)	Sensitivity, % (95% CI)	Specificity, % (95% CI)
Age < 80	≥ 8 (n = 1,087)	81.8 (75.5–86.8)	41.0 (31.2–45.2)	69.6 (48.9–87.2)	38.6 (23.2–51.3)	72.7 (67.4–76.2)	48.7 (43.2–52.4)
(n = 1,768)	≥ 9 (n = 669)	56.8 (49.0–63.6)	64.5 (54.9–68.9)	47.8 (27.2–67.1)	62.3 (45.2–73.8)	52.7 (46.6–56.5)	75.6 (70.3–78.7)
	≥ 10 (n = 394)	38.0 (30.5–44.7)	79.6 (71.6–83.2)	39.1 (19.0–57.5)	77.9 (59.6–85.6)	33.7 (28.0–37.1)	88.0 (83.6–90.2)
Age ≥ 80	≥ 8 (n = 573)	76.6 (67.8–83.9)	47.8 (34.9–53.5)	66.7 (49.3–83.1)	45.6 (29.8–56.9)	67.7 (46.1–58.4)	53.9 (60.8–72.2)
(n = 1,046)	≥ 9 (n = 351)	60.4 (50.1–68.7)	69.6 (56.5–74.4)	43.3 (26.2–61.2)	66.7 (51.1–76.7)	46.8 (39.2–51.7)	75.4 (68.2–79.3)
	≥ 10 (n = 208)	39.6 (29.6–48.9)	82.8 (72.0–86.8)	33.3 (16.5–50.7)	80.8 (64.9–87.8)	31.1 (24.4–35.6)	88.2 (82.3–91.0)

*NEWS* National early warning score, *ICU* intensive care unit, *CI* confidence interval.

### Effects of NEWS parameters on severe outcomes

**Figure 2:**
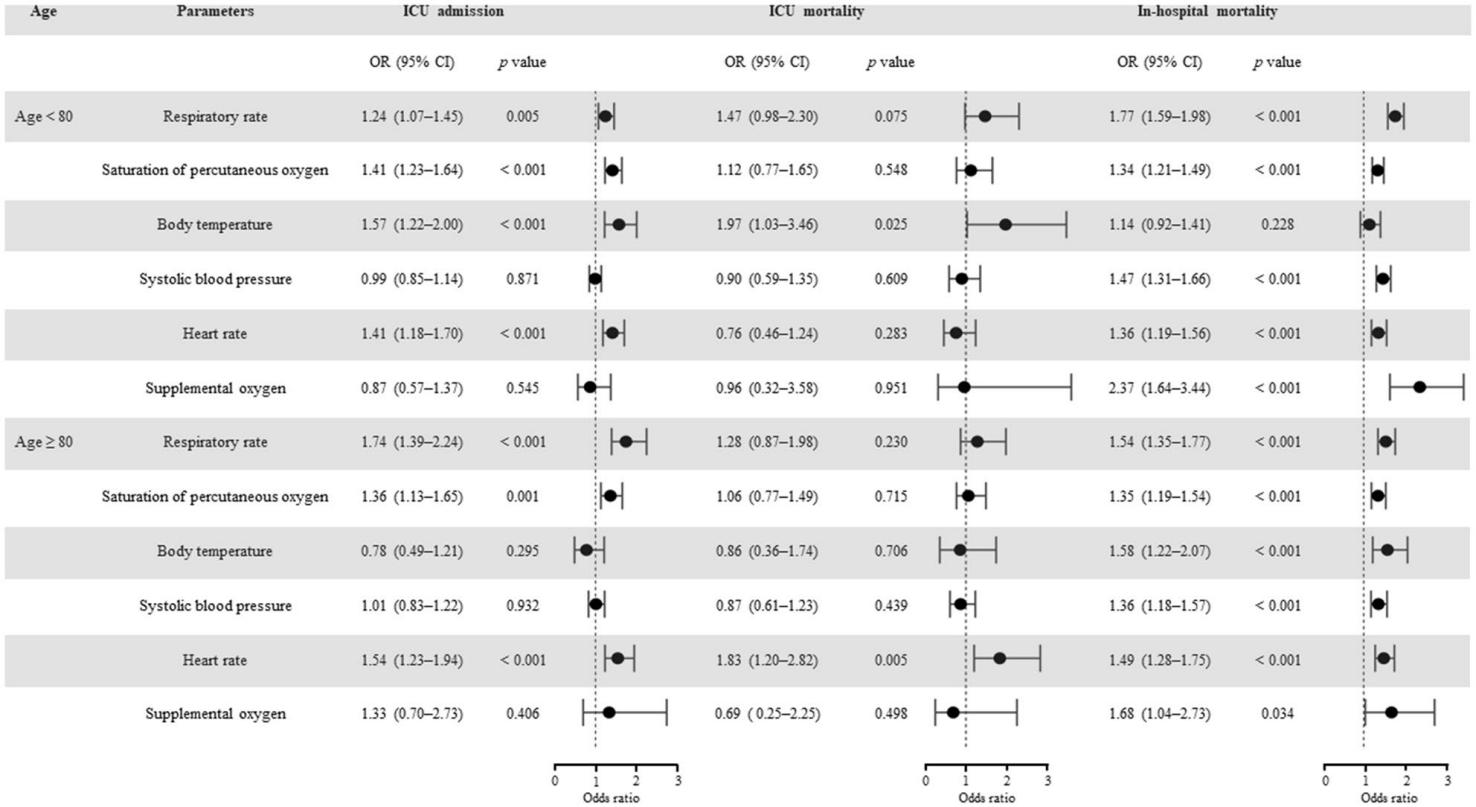
Effects of National early warning score parameters on severe outcomes by age group. *ICU* intensive care unit, *OR* odds ratio, *CI* confidence interval.

In the multivariable logistic regression analysis, body temperature was significantly related to unplanned ICU admission in patients under 80 years (odds ratio [OR] 1.57, 95% CI 1.22–2.00, *p* < 0.001), but not in patients 80 years or older (OR 0.78, 95% CI 0.49–1.21, *p* = 0.295) (Fig. 2). However, body temperature had an inverse relationship with in-hospital mortality. The results of sensitivity analyses using raw NEWS parameters are shown in Table 3.

**Table 3:** Sensitivity analysis for effects of raw National early warning parameters on severe outcomes by age group.

	NEWS parameters	Unplanned ICU admission	ICU mortality	In-hospital mortality
		Odds ratio (95% CI, *p* value)	Odds ratio (95% CI, *p* value)	Odd ratio (95% CI, *p* value)
Age < 80 (n = 1,768)	Respiratory rate	1.05 (1.01–1.08, 0.006)	1.04 (0.95–1.13, 0.347)	1.10 (1.07–1.13, < 0.001)
	Saturation of percutaneous oxygen	0.97 (0.95–0.98, < 0.001)	1.00 (0.95–1.08, 0.984)	0.95 (0.94–0.97, < 0.001)
	Body temperature	1.33 (1.10–1.61, 0.003)	1.04 (0.63–1.72, 0.890)	0.86 (0.75–0.99, 0.033)
	Systolic blood pressure	1.01 (1.00–1.02, 0.008)	1.00 (0.98–1.02, 0.910)	0.99 (0.98–0.99, < 0.001)
	Heart rate	1.01 (1.00–1.01, 0.196)	1.00 (0.98–1.02, 0.880)	1.01 (1.00–1.02, < 0.001)
	Supplemental oxygen	0.76 (0.49–1.19, 0.219)	0.95 (0.32–3.56, 0.938)	1.66 (1.16–2.39, 0.006)
Age ≥ 80 (n = 1,046)	Respiratory rate	1.10 (1.06–1.14, < 0.001)	1.03 (0.96–1.09, 0.328)	1.04 (1.01–1.07, 0.007)
	Saturation of percutaneous oxygen	0.98 (0.95–1.00, 0.069)	0.99 (0.95–1.04, 0.667)	0.94 (0.92–0.96, < 0.001)
	Body temperature	0.86 (0.64–1.14, 0.301)	0.93 (0.55–1.53, 0.773)	0.72 (0.60–0.87, < 0.001)
	Systolic blood pressure	1.01 (1.00–1.02, 0.104)	1.00 (0.99–1.02, 0.565)	0.99 (0.98–1.00, 0.009)
	Heart rate	1.01 (1.01–1.02, 0.002)	1.02 (1.01–1.04, 0.008)	1.02 (1.01–1.02, < 0.001)
	Supplemental oxygen	1.09 (0.57–2.26, 0.798)	0.65 (0.24–2.13, 0.434)	1.17 (0.73–1.88, 0.517)

*NEWS* National early warning score, *ICU* intensive care unit, *CI* confidence interval.

In subgroup analyses for patients 80 years or older, the AUROC of NEWS for unplanned ICU admission was significantly higher in patients with non-pulmonary diseases, high BMI, genitourinary disease, and neurological disease (Supplementary Table S1). In contrast, the AUROC of NEWS for in-hospital mortality was significantly higher in male, low BMI, and cancer patients. Among patients under 80 years, significant differences existed in gastrointestinal, neurological, and cancer subgroups for predicting unplanned ICU admission and BMI and genitourinary subgroups for predicting in-hospital mortality (Supplementary Table S2).

## Discussion

Our study found that NEWS had a fair predictive ability for unplanned ICU admission and in-hospital mortality among the patients identified by the RRS. There were no differences in the ability of NEWS for predicting unplanned ICU admission and in-hospital mortality between patients 80 years or older and patients under 80 years. However, the overall prediction accuracy for ICU mortality was low in both age groups, possibly due to the small ICU mortality cases.

NEWS is widely used as a reference score for RRS, and patients with NEWS seven or higher are considered at high risk of deterioration^15,20^. Smith et al. reported that NEWS outperformed other early warning scores for predicting cardiac arrest, unplanned ICU admission, and death within 24 h^21^. Liu et al. and Smith et al. also showed that NEWS had significant discriminative power for identifying in-hospital mortality^22,23^. However, little is known about indicators that predict severe outcomes in patients identified as high risk through NEWS. Our findings could provide references for managing patients identified by the RRS.

To the best of our knowledge, no previous studies have suggested additional cut-off values of the NEWS for predicting severe outcomes among elderly patients identified by the RRS. The cut-off of the NEWS discriminating high risk patients is well established^24,25^. However, in elderly populations, a large number of patients can be classified as high risk^26^. It is also challenging to perform intensive management for all these patients in resource-limited situations^27^. Therefore, additional screening criteria are necessary to assess which patients require immediate intensive care. We estimated cut-off values in high risk patients that can predict unplanned ICU admission and in-hospital mortality. The cut-off value for unplanned ICU admission was higher in patients 80 years or older. In this regard, transfer to ICU should be carefully considered in very elderly patients identified by the RRS.

Among NEWS parameters, respiratory rate, SpO2, and heart rate affected unplanned ICU admission, while all parameters affected in-hospital mortality. In particular, body temperature did not affect unplanned ICU admission in patients 80 years or older. Thermoregulation and immune function are lower in elderly populations, which can cause an afebrile condition in people with severe infection^28,29^. Consequently, body temperature may not affect unplanned ICU admission. On the other hand, body temperature had a significant effect on in-hospital mortality. In very elderly patients, body temperature changes may lead to adverse outcomes, such as in-hospital mortality. Therefore, in implementing RRS, body temperature in patients 80 years or older may need to be interpreted carefully.

In subgroup of patients with low BMI, the predictive ability of NEWS was poor for unplanned ICU admission but good for in-hospital mortality. Low BMI is associated with decreased health conditions, such as malnutrition and cachexia, relating to poor disease prognosis^30^. Among high risk patients with low BMI, NEWS may predict a long-term prognosis than a sudden deterioration. The predictive ability of NEWS for unplanned ICU admission was poor in patients 80 years or older with pulmonary diseases. A previous study reported the concern about a decreased ability of NEWS in patients with chronic hypoxemia^31^. In addition, there was an attempt to supplement respiratory parameters, such as NEWS2, which adds hypercapnic respiratory failure assessment to NEWS^32^. Therefore, a multi-dimensional approach will be required in patients 80 years or older with pulmonary diseases. Meanwhile, the predictive ability of NEWS for in-hospital mortality was superior for men. It may be due to gender differences in various diseases^33,34^. Mechanisms related to these differences, such as oxidative stress, cannot be measured with NEWS^35^. Gender differences in the prediction of severe outcomes should be evaluated in future studies.

The limitation of our study is that our findings were derived from a single-center-based retrospective cohort in South Korea. Each country has its health care system. Therefore, the results of this study may not be widely generalizable. In addition, a relatively small number of patients participated during the mid-term period. Therefore, potential selection bias should be considered in the interpretation of our findings. However, as we mentioned earlier, this study was based on a nationwide RRS pilot program. Besides, the hospital where this study was conducted is located in the state with the highest number of elderly patients in South Korea. Further validation is required after the RRS program is officially launched.

Nevertheless, our study has several strengths. A large number of patients 80 years or older participated in this study, enabling it to serve as a reference for hospitalized patient care for a growing number of aging societies. Healthcare resources can be strained due to external factors such as the coronavirus-19 (COVID-19) pandemic, and our findings can help clinicians prioritize high risk patients who need immediate intensive care. We believe that accurately applied intensive care can help prevent a pandemic-induced collapse of a healthcare system. Finally, cut-off values and important parameters for NEWS were presented. Besides, we found characteristics that NEWS performed with high accuracy in patients 80 year or older; thus, clinicians can apply NEWS when making treatment or care decisions for very elderly patients.

In conclusion, the NEWS showed fair predictive ability for unplanned ICU admission and in-hospital mortality among patients identified by RRS. Clinicians should consider the different presentations of patients 80 years or older at high risk of deterioration. More studies are required on the management of elderly patients at high risk of deterioration.

## Methods

### Study population

A single-center-based, retrospective, consecutive cohort study was conducted at Hallym University Chuncheon Sacred Heart Hospital, a teaching hospital, from June 2019 to December 2020. In this hospital, the RRS pilot program was operated by the Korean government^36^. When a patient’s NEWS was recorded seven or higher, the detected patient was classified as a high risk. After that, a member of the rapid response team visited the patients and intervened in their management. The RRS activations for the same patients detected at different times were regarded as separate cases. For all hospitalized patients, the NEWS was measured and recorded automatically using an electronic health-record-based system. We retrospectively reviewed all data of RRS activations, and 3,163 RRS activations from 935 patients were obtained. Of the 3,163 activations, we excluded 14 activated after ICU admission, 10 activated for patients < 18 years, and 325 with missing data. Therefore, 2,814 RRS activations from 851 patients were included in the final analysis.

### Ethics declarations

This retrospective cohort study was conducted in accordance with the Helsinki declaration and approved by the Institutional Review Board (IRB) of the Hallym University Chuncheon Sacred Heart Hospital (IRB number: 2021-02-007). The need for written informed consent was waived and confirmed by the IRB of the Hallym University Chuncheon Sacred Heart Hospital (IRB number: 2021-02-007) because this was a retrospective cohort study.

### National early warning score

The NEWS was used as a reference value for RRS activation. The cut-off value of NEWS for activating RRS was seven. The NEWS consists of seven physiological parameters, and the total score is calculated by summing scores for each parameter (Supplementary Table S3)^37^. The physiologic NEWS parameters were respiratory rate, systolic blood pressure, level of consciousness, supplemental oxygen, heart rate, saturation of percutaneous oxygen (SpO2), and body temperature. These items were scored from 0 to 3 points each, except SpO2 ranged from 0 to 2 points.

### Severe outcomes

We regarded unplanned ICU admission, ICU mortality, and in-hospital mortality as severe outcomes. Unplanned ICU admission, the main outcome of this study, was defined as abrupt transfer to the ICU within 24 h after the RRS activation. Secondary outcomes were ICU and in-hospital mortality, defined as death events within 24 h after ICU admission and within the hospitalized period, respectively. Cases of death in the ICU were also included in both ICU and in-hospital mortality. All incidents were identified automatically using the hospital’s electronic medical records.

### Other measurements

BMI was defined as body weight divided by height squared (kg/m^2^). The reason for hospital admission consisted of cardiovascular, pulmonary, gastrointestinal, genitourinary, neurological diseases, and cancer. We used international disease classifications at admission for defining the disease classification^38^. Diseases not included in the above six categories, such as severe sepsis, were classified as other diseases.

### Statistical analyses

All statistical analyses were performed using R version 4.0.3 (R core Team 2020; R Foundation for Statistical Computing, Vienna, Austria). We analyzed patients identified by the RRS according to the patient’s age. Among the NEWS parameters, continuous variables were categorized according to their scores and expressed as numbers (percentages). The *p*-value for comparing baseline characteristics was analyzed using Pearson’s chi-square test, except for the level of consciousness, which was analyzed using Fisher’s exact test. For assessing the predictive ability of the NEWS, a receiver operating characteristic (ROC) curve was drawn using the ‘pROC’ package for R. The 95% CI for the AUROC and *p*-values were calculated using Delong’s test. Sensitivity and specificity were calculated using 2000 stratified bootstrap replicates. The NEWS cut-off values for severe outcomes were calculated using the Youden index^39^.

We further analyzed the effect of NEWS parameters on severe outcomes using multivariable logistic regression analysis. Each parameter was adjusted for other parameters and the reason for hospital admission. Scored NEWS values were used for the main analysis, and sensitivity analyses were performed using the raw NEWS values. Because its observed values were low, the level of consciousness was excluded from the analysis.

Subgroup analyses were performed on sex, BMI, and the reason for hospital admission by age groups. The BMI subgroup was divided by the median. Subgroup analysis for ICU mortality was not performed due to the low number of ICU mortality cases. An additional subgroup analysis was performed with patients under 80 years. For all analyses in this study, *p*-values < 0.05 were considered statistically significant.

## Supplementary Information

Supplementary Information 1.

Supplementary Information 2.

Supplementary Information 3.

Supplementary Information 4.

## Data Availability

Our datasets are available from the corresponding author on reasonable request.

## Abbreviations

NEWS: National early warning score
ICU: Intensive care unit
RRS: Rapid response system
SpO2: Saturation of percutaneous oxygen
BMI: Body mass index
ROC: Receiver operating characteristic
CI: Confidence interval
AUROC: Area under the receiver operating characteristic curve
OR: Odds ratio
SD: Standard deviation
COVID-19: Coronavirus-19
