# Supplementary figures and images for: Predicting severe outcomes using national early warning score (NEWS) in patients identified by a rapid response system: a retrospective cohort study
Source: Sci Rep. 2021 Sep 9;11:18021. doi: 10.1038/s41598-021-97121-w (PMC8429773; doi:10.1038/s41598-021-97121-w)

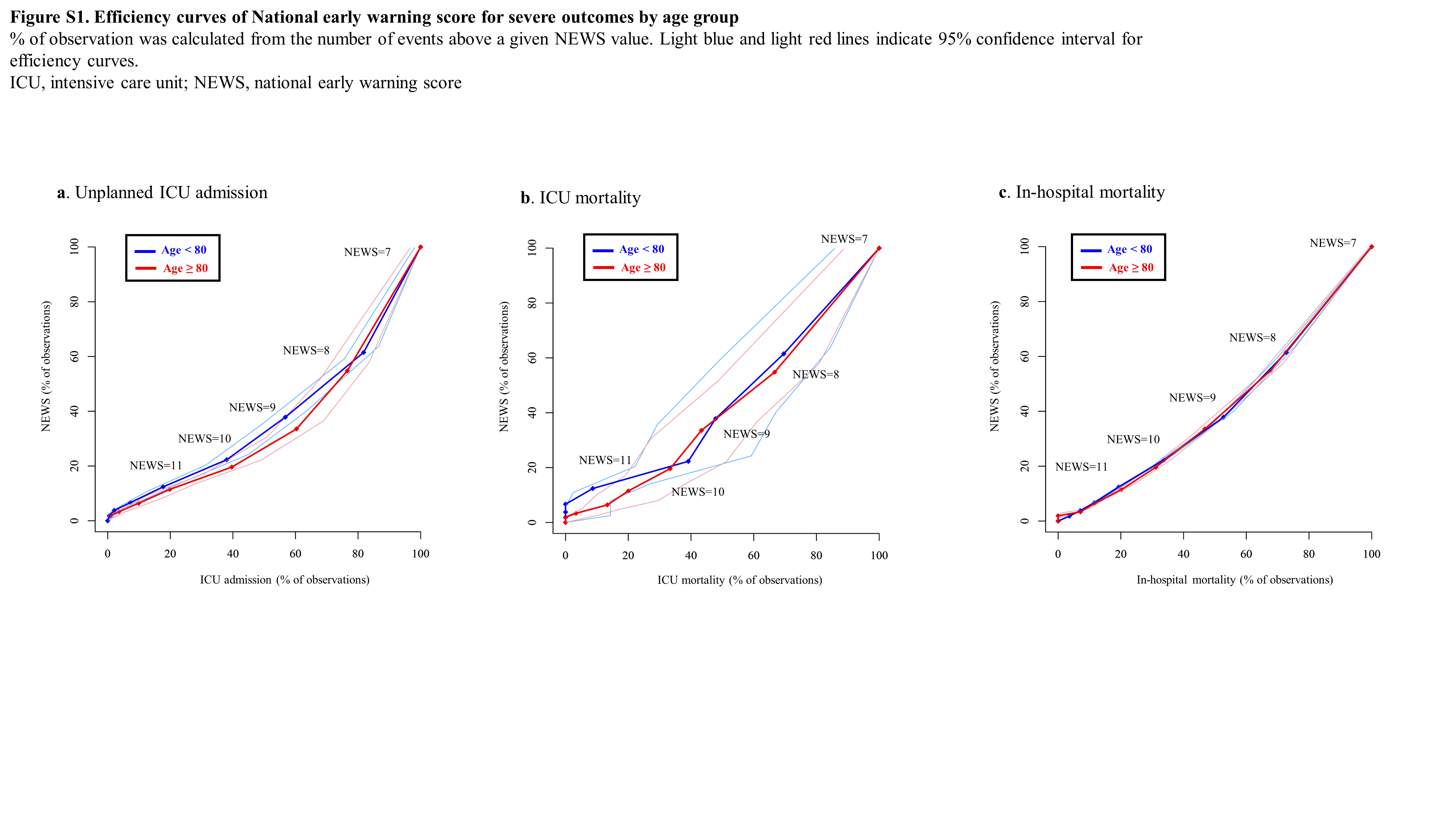

Supplement: Supplementary file 1 — Supplementary Information 1. [file 41598_2021_97121_MOESM1_ESM.tif]
