# Supplementary material for: Predicting severe outcomes using national early warning score (NEWS) in patients identified by a rapid response system: a retrospective cohort study
Source: Sci Rep. 2021 Sep 9;11:18021. doi: 10.1038/s41598-021-97121-w (PMC8429773; doi:10.1038/s41598-021-97121-w)
Supplement: Supplementary file 2 — Supplementary Information 2. [file 41598_2021_97121_MOESM2_ESM.docx]

|  |  | Unplanned ICU admission |  | In-hospital mortality |  |
| --- | --- | --- | --- | --- | --- |
| Subgroup |  | AUROC (95% CI) | *p* value | AUROC (95% CI) | *p* value |
| Sex | Male (n=585) | 0.63 (0.56–0.70) | 0.152 | 0.69 (0.64–0.73) | 0.008 |
|  | Female (n=461) | 0.71 (0.63–0.78) |  | 0.60 (0.54–0.65) |  |
| Body mass index (kg/m^2^) | <21.5 (n=535) | 0.60 (0.52–0.68) | 0.010 | 0.68 (0.63–0.72) | 0.039 |
|  | ≥21.5 (n=511) | 0.74 (0.67–0.80) |  | 0.61 (0.56–0.66) |  |
| Cardiovascular disease | Yes (n=97) | 0.66 (0.48–0.85) | 0.989 | 0.62 (0.49–0.76) | 0.752 |
|  | No (n=949) | 0.67 (0.61–0.72) |  | 0.65 (0.61–0.68) |  |
| Pulmonary disease | Yes (n=448) | 0.58 (0.50–0.66) | 0.002 | 0.64 (0.59–0.69) | 0.590 |
|  | No (n=598) | 0.74 (0.68–0.80) |  | 0.65 (0.61–0.70) |  |
| Gastrointestinal disease | Yes (n=78) | 0.71 (0.55–0.86) | 0.602 | 0.65 (0.52–0.78) | 0.969 |
|  | No (n=968) | 0.66 (0.61–0.72) |  | 0.65 (0.62–0.68) |  |
| Genitourinary disease | Yes (n=111) | 0.86 (0.76–0.96) | <0.001 | 0.60 (0.48–0.73) | 0.503 |
|  | No (n=935) | 0.65 (0.58–0.69) |  | 0.65 (0.62–0.68) |  |
| Neurological disease | Yes (n=40) | 0.85 (0.73–0.98) | 0.008 | 0.54 (0.35–0.73) | 0.265 |
|  | No (n=1,006) | 0.66 (0.60–0.71) |  | 0.65 (0.62–0.69) |  |
| Cancer | Yes (n=126) | 0.68 (0.45–0.91) | 0.973 | 0.80 (0.73–0.87) | <0.001 |
|  | No (n=920) | 0.68 (0.62–0.73) |  | 0.62 (0.58–0.66) |  |

**Table S1. Subgroup analysis for the predictive ability of National early warning score for severe outcomes in patients 80 years or older**

ICU, intensive care unit; AUROC, area under the receiver operating characteristics curve; CI, confidence interval
