# Supplementary material for: Predicting severe outcomes using national early warning score (NEWS) in patients identified by a rapid response system: a retrospective cohort study
Source: Sci Rep. 2021 Sep 9;11:18021. doi: 10.1038/s41598-021-97121-w (PMC8429773; doi:10.1038/s41598-021-97121-w)
Supplement: Supplementary file 3 — Supplementary Information 3. [file 41598_2021_97121_MOESM3_ESM.docx]

|  |  | ICU admission |  | In-hospital mortality |  |
| --- | --- | --- | --- | --- | --- |
| Subgroup |  | AUROC (95% CI) | *p* value | AUROC (95% CI) | *p* value |
| Sex | Male (n=1,110) | 0.64 (0.59–0.68) | 0.803 | 0.66 (0.63–0.69) | 0.693 |
|  | Female (n=658) | 0.65 (0.58–0.71) |  | 0.67 (0.63–0.71) |  |
| Body mass index (kg/m^2^) | <21.5 (n=818) | 0.63 (0.58–0.69) | 0.846 | 0.69 (0.66–0.73) | 0.044 |
|  | ≥21.5 (n=950) | 0.64 (0.59–0.70) |  | 0.64 (0.61–0.68) |  |
| Cardiovascular disease | Yes (n=1,691) | 0.61 (0.40–0.82 | 0.795 | 0.77 (0.65–0.88) | 0.078 |
|  | No (n=77) | 0.64 (0.60–0.68) |  | 0.66 (0.64–0.69) |  |
| Pulmonary disease | Yes (n=1,168) | 0.66 (0.59–0.73) | 0.471 | 0.66 (0.62–0.70) | 0.668 |
|  | No (n=600) | 0.63 (0.58–0.68) |  | 0.67 (0.64–0.70) |  |
| Gastrointestinal disease | Yes (n=134) | 0.74 (0.65–0.83) | 0.044 | 0.65 (0.54–0.75) | 0.751 |
|  | No (n=1,634) | 0.63 (0.59–0.67) |  | 0.67 (0.64–0.69) |  |
| Genitourinary disease | Yes (n=129) | 0.61 (0.49–0.72) | 0.519 | 0.49 (0.38–0.61) | 0.003 |
|  | No (n=1,639) | 0.65 (0.61–0.69) |  | 0.68 (0.65–0.70) |  |
| Neurological disease | Yes (n=133) | 0.90 (0.83–0.96) | <0.001 | 0.64 (0.47–0.81) | 0.782 |
|  | No (n=1,635) | 0.61 (0.57–0.65) |  | 0.67 (0.64–0.69) |  |
| Cancer | Yes (n=1,228) | 0.54 (0.43–0.65) | 0.019 | 0.69 (0.65–0.74) | 0.052 |
|  | No (n=540) | 0.68 (0.64–0.72) |  | 0.64 (0.61–0.67) |  |

**Table S2. Subgroup analysis for the predictive ability of National early warning score for severe outcomes in patients under 80 years**

AUROC, area under the receiver operating characteristics curve; CI, confidence interval
