# Supplementary material for: Predicting severe outcomes using national early warning score (NEWS) in patients identified by a rapid response system: a retrospective cohort study
Source: Sci Rep. 2021 Sep 9;11:18021. doi: 10.1038/s41598-021-97121-w (PMC8429773; doi:10.1038/s41598-021-97121-w)
Supplement: Supplementary file 4 — Supplementary Information 4. [file 41598_2021_97121_MOESM4_ESM.docx]

| Parameters | 3 | 2 | 1 | 0 | 1 | 2 | 3 |
| --- | --- | --- | --- | --- | --- | --- | --- |
| Respiratory rate (rates/min) | ≤8 |  | 9-11 | 12-20 |  | 21-24 | ≥25 |
| Saturation of percutaneous oxygen (%) | ≤91 | 92-93 | 94-95 | ≥96 |  |  |  |
| Oxygen supplement |  | Yes |  | No |  |  |  |
| Body temperature (℃) | 35.5 |  | 35.1-36.0 | 36.1-38.0 | 38.1-39.0 | ≥39.1 |  |
| Systolic Blood pressure (mmHg) | ≤90 | 91-100 | 101-110 | 111-219 |  |  | ≥220 |
| Heart rate (beats/min) | ≤40 |  | 41-50 | 51-90 | 91-110 | 111-130 | ≥131 |
| Level of consciousness |  |  |  | A |  |  | V, P, or U |

**Table S3. National early warning score**

A, awake; V, verbal; P, pain; U, unresponsive
